# Descriptive analytical frameworks for benefit-risk assessment in health technology assessment

**DOI:** 10.1590/S2237-96222026v35e20251399.en

**Published:** 2026-06-12

**Authors:** Sidney Marcel Domingues, Patrícia Coelho de Soárez, Erica Aranha Suzumura, Ana Carolina Nonato, Aline Frossard Ribeiro Bortoluzzi, Fernando Henrique de Albuquerque Maia, Bruna de Oliveira Ascef, Beate Jahn, Uwe Siebert

**Affiliations:** 1 Universidade de São Paulo, Faculdade de Medicina, São Paulo, SP, Brazil Universidade de São Paulo Faculdade de Medicina São Paulo SP Brazil; 2 UMIT TIROL - University for Health Sciences and Technology, Institute of Public Health, Medical Decision Making and Health Technology Assessment, Hall in Tirol, Tyrol, Austria UMIT TIROL - University for Health Sciences and Technology Institute of Public Health, Medical Decision Making and Health Technology Assessment Hall in Tirol Tyrol Austria; 3 ONCOTYROL - Center for Personalized Cancer Medicine, Division of Health Technology Assessment, Innsbruck, Tyrol, Austria ONCOTYROL - Center for Personalized Cancer Medicine Division of Health Technology Assessment Innsbruck Tyrol Austria; 4 Harvard T.H. Chan School of Public Health, Center for Health Decision Science and Departments of Epidemiology and Health Policy & Management, Boston, MA, United States Harvard T.H. Chan School of Public Health Center for Health Decision Science and Departments of Epidemiology and Health Policy & Management Boston MA United States; 5 Harvard Medical School, Mass General Brigham Center for Health Technology Assessment, Boston, MA, United States Harvard Medical School Mass General Brigham Center for Health Technology Assessment Boston MA United States

Analytical frameworks used in benefit-risk assessment are conceptual frameworks that support decision-making and are generally categorized as descriptive or quantitative. In this article, we present the main descriptive analytical frameworks for benefit-risk assessment that can be applied in the context of health technology assessment.

## What are analytical frameworks?

Although there is no universal definition of an analytical framework, the term generally refers to the conceptual framework that brings together assumptions, values and practices that establish connections between the scientific and political domains. Its main function is to provide a common language that guides which concepts and which fundamental relationships should be measured and compared in empirical research^1^.

## Analytical frameworks for benefit-risk analysis

In the context of benefit-risk assessment, analytical frameworks enable a systematic, explicit, transparent and reproducible approach to assessing and communicating the balance between the main benefits and risks (or harms) of a technology, along with their associated uncertainties.

Descriptive analytical frameworks are structures with qualitative instructions that provide guidance right from planning benefit-risk assessment though to the moment a final judgment on benefit-risk balance is made. They can be used to select, organize, summarize and communicate the rationale for decisions. A descriptive analytical framework is the indispensable basis of any benefit-risk assessment^2^.

The following stand out among the main descriptive analytical frameworks for benefit-risk assessment: the Benefit-Risk Action Team (BRAT) framework; the Problem, Objectives, Alternatives, Consequences, Trade-offs, Uncertainty, Risk and Linked Decisions (PrOACT-URL) framework; and the US Food and Drug Administration - Benefit-Risk Framework (FDA BRF)^3^^), (^^4^. PrOACT-URL and FDA BRF were developed by regulatory agencies. The European Medicines Agency (EMA), for example, is responsible for the final marketing authorization of medicinal products for the European Union. Only after this authorization does the health technology assessment process begin.

### Benefit-Risk Action Team (BRAT) framework

The BRAT analytical framework was developed by Pharmaceutical Research and Manufacturers of America (PhRMA) in collaboration with several associated companies^5^. It was created based on a literature review of existing benefit-risk assessment methods, which showed that none of the available methods included all the desirable criteria - ease of use, update capability, integration of multiple benefits and risks, flexibility for application in various therapeutic areas and ability to incorporate the preferences of stakeholders such as patients, physicians and regulators^6^.

The BRAT framework was developed to improve benefit-risk assessment and to recommend a transparent and systematic approach to support decision-making. This analytical framework assesses benefits and risks separately, facilitating their comparison and avoiding synthesis of data into complex statistical models that can be difficult for readers to understand.

Between 2008 and 2009, BRAT was enhanced in collaboration with epidemiologists, tested in simulated cases and assessed by regulatory and clinical experts. Developed specifically for benefit-risk assessment of pharmaceutical products, the framework is based on consolidated methodologies, such as systematic reviews of evidence and meta-analyses and multi-criteria decision analysis^7^.

The BRAT framework has the following characteristics: it places emphasis on building the value tree, data selection and preparation; it assesses benefits and risks separately, not integrated into the framework; it can incorporate the weighting of results, without focusing on the calculation of overall benefit-risk scores^6^^), (^^7^^), (^^8^.

It is applied in decision-making and in communicating benefit-risk assessment between pharmaceutical companies and regulatory bodies through a process carried out in six steps^6^^), (^^7^^), (^^8^ (Table 1).

**Table 1:** Benefit-Risk Action Team (BRAT) analytical framework steps

Step	Description
**1. Define the decision context**	• Define the drug, dose, formulation, indication, patient population, comparator(s), time horizon for outcomes, and the perspective of the decision-makers (regulator, company, patient or physician).
**2. Identify outcomes**	• Select all the important outcomes and create the initial value tree.
	• Define a preliminary set of metrics for each outcome.
	• Document the rationale for the included/excluded outcomes.
**3. Identify and extract data source**	• Determine and document all data sources (e.g., clinical trials, observational studies). Extract all relevant data for the data source table, including detailed references and any annotations, to aid in subsequent interpretations and create summary metrics.
**4. Customize the framework**	• Modify the value tree based on further data review and clinical expertise.
	• Refine the outcome metrics. This may include adjustments to outcomes that are not considered relevant to a specific benefit-risk assessment or that vary in relevance depending on the stakeholder group.
**5. Assess outcome importance**	• Rank or weight of the importance of outcomes for decision-makers or other stakeholders.
**6. Display and interpret key benefit-risk metrics**	• Summarize the data in tabular and graphical displays to aid in review and interpretation.
	• Question the summary metrics, review the data sources and identify and complete any information gaps.
	• Interpret the summarized information.

### Problem, Objectives, Alternatives, Consequences, Trade-offs, Uncertainty, Risk and Linked decisions (PrOACT-URL)

The PrOACT-URL framework was developed by the EMA in collaboration with the London School of Economics and the University of Groningen, as part of the EMA Benefit-Risk Methodology Project, between 2010 and 2011^9^. This project reviewed recognized methods for benefit-risk assessment and those presented in the reports of the Committee for Medicinal Products for Human Use (CHMP), suggesting incorporation of specific criteria and guidelines on benefits and risks, as well as development of methodologies for systematic analysis of the benefit-risk balance^9^.

PrOACT-URL follows the PrOACT decision-making methodology, developed by decision theory scientists^10^. It was developed to improve the transparency, consistency and communication of benefit-risk assessment, being specifically targeted at regulatory decision-making on medicinal products^9^.

Its qualitative approach is sufficient for most cases. In more complex situations, however, its structure has been used as a basis for other methodologies^11^, because it allows integration with other quantitative methodologies, such as decision analytic models and multi-criteria decision analysis^12^.

PrOACT-URL allows the use of different judgments and sources by different stakeholders, such as drug developers, regulatory bodies, health technology assessment bodies, healthcare providers and patients^13^. Its flexibility is such that its implementation in benefit-risk assessment can extend to all stages of a health technology life cycle, which demonstrates its applicability to the field of health technology assessment^3^. A quantitative model developed by a regulator can be reused by the health technology assessment body, which can add cost and QALY criteria, as well as calculating the incremental cost-effectiveness ratio^14^.

PrOACT-URL^13^^), (^^15^ is performed in 12 steps (Table 2). Step six suggests creating an effects table, which presents all the favorable and unfavorable effects that assessors take into consideration to influence the balance between benefits and risks, along with comments on uncertainties that, in the assessor’s view, persist in the assessment^13^.

**Table 2:** Problem, Objectives, Alternatives, Consequences, Trade-offs, Uncertainty, Risk, and Linked decisions (PrOACT-URL) analytical framework steps

Step	Description
**Problem**	
1. Determine the nature of the problem and its context	• The technology (e.g., new drug or biologic, device, generic, marketed).
	• Indication(s) for use.
	• Disease treatment area and epidemiology.
	• Unmet medical need, condition severity and morbidity, affected population, patient and physician concerns, health outcome time frame.
	• The decision problem (what needs to be decided and by whom, e.g., industry, regulator, healthcare provider, patient).
2. Structure the problem	• Whether this is primarily a problem of uncertainty, or of multiple conflicting objectives, or a combination of both (e.g., temporal progression of health states).
	• Factors to be considered in solving the problem (e.g., study design, data sources and adequacy, disease epidemiology, alternative treatment availability).
**Objetives**	
3. Establish objectives for the overall goals to be achieved	• Objective (e.g., assess the benefit-risk balance, determine what additional information is needed, evaluate the change in the benefit-risk balance, recommend restrictions).
4. Identify criteria for: a) Favorable effects; b) Unfavorable effects	• A complete set of criteria that includes both favorable and unfavorable effects (e.g., outcomes, relevant health states, clinical results). An operational definition for each criterion along with a defined two-point measurement scale to encompass the performance range of the alternatives (not just reported measures of central tendency, but also confidence intervals). Considerations regarding the clinical relevance of the criteria - some are of greater concern to decision-makers than others.
**Alternatives**	
5. Identify the objectives to be assessed in relation to the criteria	• Pre-approval: dosage, treatment timing, drug vs. placebo and/or active comparator; the necessary decision or recommendation (e.g., approve/disapprove, restrict, withdraw).
	• Post-approval: do nothing, limit duration, restrict indication, suspend.
**Consequences**	
6. Describe how the alternatives behave for each of the criteria, that is, the magnitudes of all effects, severity, and incidence of all effects	• The consequences are presented separately for each alternative in each criterion (e.g., clinically relevant efficacy and safety effects, positive and negative health outcomes), summarized in an effects table with alternatives in columns and criteria in rows. Qualitative and quantitative descriptions of the effects in each table cell are provided, including statistical summaries with confidence intervals and references to the source data, graphs, and diagrams.
**Trade-offs**	
7. Assess the balance between the favorable and unfavorable effects	• The judgment regarding the benefit-risk balance and the rationale for the judgment.
**Up to this step, only issues related to favorable and unfavorable effects, and their balance, have been considered. The next three steps are relevant for considering how the benefit-risk balance is affected when taking uncertainties into account.**	
**Uncertainty**	
8. Report the uncertainty associated with the favorable and unfavorable effects	• The basis and extent of uncertainty beyond statistical probabilities (e.g., potential data biases, robustness and representativeness of clinical trials, potential for unobserved adverse effects).
9. Consider how the balance between favorable and unfavorable effects is affected by uncertainty	• The extent to which the benefit-risk balance in step seven is reduced when considering all sources of uncertainty, in order to provide a benefit-risk balance, and the reasons for the reduction.
**Risk attitudes**	
10. Assess the relative importance of the attitude of the decision-maker in relation to the risk for this product.	• Any consideration that may or should affect the decision-maker’s attitude toward the risk for this product (e.g., orphan drug status, special population, unmet medical need, risk management plan).
11. Report how this affected the balance reported in step 9	• The basis for the decision-maker’s assessment of how tolerable the benefit-risk balance is considered to be (taking into account stakeholders’ views on the risk).
**Linked decisions**	
12. Consider the consistency of this decision with similar past decisions and assess whether making this decision may impact future decisions	• How this decision, and the value judgments and data on which it is based, might set a precedent or make similar decisions in the future easier or more difficult.

It is important to emphasize that both analytical frameworks, BRAT and PrOACT-URL, are based on well-established decision analysis principles^6^. The common roots of the methods result in many similarities and a close relationship between them. The main difference is that the last two steps of the ProACT-URL analytical framework consider broader issues, such as the decision-maker’s risk attitudes and consistency with similar previous decisions, issues that are not formal parts of the BRAT framework^15^.

### US Food and Drug Administration - Benefit-Risk Framework (FDA BRF)

The FDA BRF was developed by the United States Food and Drug Administration (FDA) and its implementation began in 2013^12^. This analytical framework provides a structured approach to benefit-risk assessment of drugs and biological products, taking into account issues relevant to regulatory decision-making and internal communication to the FDA and the public^16^.

Benefit-risk assessment using FDA BRF does not end with the approval of a drug, but continues throughout its entire life cycle, as benefits and risks can change over time based on new information about its efficacy or safety. In the post-marketing period, the benefit-risk balance is reassessed based on scientific literature, post-marketing studies, reports of adverse events, medication errors, product quality and patient experience data^16^.

The FDA BRF recognizes the importance of incorporating patient input into drug development and regulatory decisions, whereby taking into consideration their experiences and perspectives is essential for understanding the impact of the disease, the most relevant benefits and acceptance of risks throughout the drug’s lifecycle^16^.

The FDA BRF^16^ offers clear and easy-to-apply guidelines. It analyzes the decision dimensions, which include condition analysis, current treatment options, benefits, risks and risk management. It also considers evidence and uncertainties related to these factors, which lead to conclusions and underlying reasons, i.e., implications for decision-making (Table 3).

**Table 3:** Description of the decision factors, evidence, uncertainties, conclusions and rationale for benefit-risk assessment, using the US Food and Drug Administration - Benefit-Risk Framework (FDA BRF)

Dimensions	Important considerations	Conclusions and rationale
**Analysis of condition**	• Proposed indication use context: intended medical use, target patient population, aspects of the condition that the treatment seeks to address.	• Conclusions (implications for decision)
	• Most relevant aspects of the condition indicated.	
	• Public health implications of the disease.	
**Current treatment options**	• Understanding of current treatment approved by the FDA and of the care standard.	• Conclusions (implications for decision)
	• Efficacy and safety of other interventions available for the target population.	
	• Patient’s medical need for new treatment.	
**Benefit**	• Clinical trial strengths/limitations, including their design, and their possible implications for assessing drug efficacy.	• Conclusions (implications for decision)
	• Study outcome clinical relevance: ability to measure or predict clinical results important for patients.	
	• Description of the clinical benefits, including but not limited to:	
	• Nature of the effect (e.g., survival, slowing of disease progression, reduction of serious outcomes, reduction of symptoms, relevance of symptomatic benefit for patients).	
	• Magnitude of the effect and associated uncertainty (e.g., confidence interval), together with an interpretation of its clinical relevance.	
	• Distribution of treatment effects in the clinical trial population (e.g., presence of patients experiencing a more substantial benefit, such as prolonged survival or marked improvement in symptoms, even if the average response is modest).	
	• Temporal evolution and durability of the effect.	
	• Benefit attributed to the drug when studied in combination with other treatments.	
	• Subpopulations achieving greater magnitude of benefit defined.	
	• Benefit for a subpopulation with a specific unmet medical need (e.g., patients who have not responded adequately to available treatments).	
	• Generalization of demonstrated benefits for all populations likely to receive the drug (e.g., elderly patients or those with comorbidities not extensively studied in clinical trials).	
	• Important characteristics of the drug (e.g., less onerous dosage regimen, method of dose preparation, or administration route).	
**Risk and risk management**	• Strengths and limitations of the safety evidence, and its potential implications for assessing the risks of the drug (e.g., due to limited database size and/or duration of exposure, or absence of important subpopulations).	• Conclusions (implications for decision)
	• Adverse events or safety signs observed and their clinical relevance, including:	
	• Adverse event severity, probability of occurrence, reversibility, and estimate of the magnitude of the effect and its uncertainty (e.g., confidence interval).	
	• Ability to predict, monitor and/or prevent adverse event.	
	• Impact of adverse events on drug tolerability and/or adherence, and possible consequences.	
	• Level of certainty regarding the causal association between exposure to the drug and risk.	
	• Potential impact of product quality issues that could negatively affect the safety or efficacy of the drug, including quality considerations that would need to be monitored post-marketing.	
	• Expected differences in safety in the post-marketing period, compared to the clinical trial setting (e.g., due to lower likelihood of adequate monitoring or use in patients at higher risk of adverse events).	
	• Potential for misuse or accidental exposure, and associated adverse consequences.	
	• Probable effectiveness of the proposed approaches for managing these risks.	
**Conclusion regarding benefit-risk balance**		
• General conclusions regarding the quality and robustness of the evidence, as well as remaining uncertainties concerning benefits and risks.		
• Influence of the treatment context on assessment of benefits, risks and uncertainties.		
• Relative importance of benefits and risks in the general target population, also considering the perspectives of patients.		
• Temporal evolution of benefits and risks (e.g., taking into account adverse events that may occur soon after the start of treatment as opposed to benefits that may take years to manifest themselves).		
• Ability of patients and healthcare professionals to clearly assess the benefits or risks of a drug (e.g., by assessing symptom relief or monitoring changes in biomarkers), thus contributing to informed treatment decisions (e.g., discontinuing the drug if there is no adequate response).		

## Applying descriptive analytical frameworks for benefit-risk assessment in the context of health technology assessment

Use of descriptive analytical frameworks in benefit-risk assessment has been observed over the last three decades (2000s, 2010s, 2020s), with a view to applying more structured, objective and transparent approaches, resulting in improved communication and decision-making^3^^), (^^11^^), (^^12^^), (^^17^. In the context of health technology assessment, according to a scoping review published in 2024, the most cited descriptive analytical frameworks were PrOACT-URL and BRAT. The most recommended were PrOACT-URL and FDA BRF^4^.

BRAT, PrOACT-URL and FDA BRF share common characteristics, such as practicality, consistency, transparency, systematic approach and decision context. They also share quality and interpretation of evidence, transparency of prioritization and weighting of benefits and risks, and monitoring of uncertainties. Finally, all three share assessment, summarization and communication of relevant data and information through visual tools such as value trees or effects tables, which will be discussed in the final article of this series^3^.

The FDA continues to use a descriptive analytical framework for benefit-risk assessment, despite recognizing the potential of quantitative methods. The EMA has moved forward in adopting quantitative analytical frameworks^18^, which will be discussed in the next article in this series.

It is important to highlight that, in cases in which a new technology increases benefits and reduces risks, or when risks clearly outweigh benefits, applying a descriptive analytical framework is sufficient to determine the benefit-risk balance of that technology^19^. Recently, the Grading of Recommendations Assessment, Development and Evaluation (GRADE) Guidance established the use of decision thresholds that can support judgments about the balance between benefits and risks (harms) in health technology assessment^20^, which will be presented in the penultimate article of this series.

In the context of health technology assessment in Brazil, implementing these analytical frameworks can represent an opportunity to further improve decision-making by bodies such as CONITEC, especially by making the criteria and uncertainties involved in recommendations more explicit. However, the challenges include the need for technical training, adaptation to the institutional and regulatory specificities of the Brazilian Unified Health System, and integration with current assessment and social participation processes.

Gene therapies and, in general, technologies aimed at treating rare diseases are good examples that can be the subject of technical training. These technologies require special attention by regulatory and health technology assessment agencies, since benefit-risk assessment tends to be more complex and to have a high degree of uncertainty due to the scarcity of data, the small number of patients, as well as methodological limitations of available studies.

It is recommended that comprehensive health technology assessment studies consider including at least one structured descriptive analytical framework^17^, in order to transparently present the value judgments and rationale that supported decision-making, ensuring that the main aspects of the assessment process have not been neglected. The evidence-based balance between benefits and risks (harms) should also be explicitly communicated in decision-making processes.

## Data Availability

The data are available within the body of the manuscript.
