# Supplementary material for: Descriptive analytical frameworks for benefit-risk assessment in health technology assessment
Source: Epidemiol Serv Saude. 2026 Jun 12;35:e20251399. doi: 10.1590/S2237-96222026v35e20251399.en (PMC13686306; doi:10.1590/S2237-96222026v35e20251399.en)
Supplement: Translation

# Translation: Estruturas analíticas descritivas para avaliação benefício-risco na avaliação de tecnologias em saúde

**DOI:** 10.1590/S2237-96222026v35e20251399.pt

**Authors:** Sidney Marcel Domingues, Patrícia Coelho de Soárez, Erica Aranha Suzumura, Ana Carolina Nonato, Aline Frossard Ribeiro Bortoluzzi, Fernando Henrique de Albuquerque Maia, Bruna de Oliveira Ascef, Beate Jahn, Uwe Siebert

**Affiliations:** 1 Universidade de São Paulo, Faculdade de Medicina, São Paulo, SP, Brasil; 2 UMIT TIROL - University for Health Sciences and Technology, Institute of Public Health, Medical Decision Making and Health Technology Assessment, Hall in Tirol, Tirol, Áustria; 3 ONCOTYROL - Center for Personalized Cancer Medicine, Division of Health Technology Assessment, Innsbruck, Tirol, Áustria; 4 Harvard T.H. Chan School of Public Health, Center for Health Decision Science and Departments of Epidemiology and Health Policy & Management, Boston, MA, Estados Unidos; 5 Harvard Medical School, Mass General Brigham Center for Health Technology Assessment, Boston, MA, Estados Unidos

As estruturas analíticas (frameworks) utilizadas na avaliação benefício-risco constituem estruturas conceituais que apoiam a tomada de decisão e são geralmente categorizadas em descritivas ou quantitativas. Neste artigo, apresentamos as principais estruturas analíticas descritivas para avaliação benefício-risco que podem ser aplicadas no contexto de avaliação de tecnologias em saúde.

## O que são estruturas analíticas?

Embora não haja uma definição universal de estrutura analítica, o termo refere-se, em geral, a estrutura conceitual que integra pressupostos, valores e práticas que estabelece conexões entre o domínio científico e o político. Sua principal função consiste em fornecer uma linguagem comum que orienta quais conceitos e quais relações fundamentais devem ser mensuradas e comparadas nas pesquisas empíricas^1^.

## Estruturas analíticas para avaliação benefício-risco

No contexto da avaliação benefício-risco, estruturas analíticas possibilitam uma abordagem sistemática, explícita, transparente e reprodutível para avaliar e comunicar o balanço entre os principais benefícios e riscos (ou danos) de uma tecnologia, com as incertezas associadas.

Estruturas analíticas descritivas são arcabouços com instruções qualitativas que orientam desde o planejamento da avaliação benefício-risco até o momento em que um julgamento final sobre o balanço benefício-risco é realizado. Elas podem ser utilizadas para selecionar, organizar, resumir e comunicar as justificativas das decisões. A estrutura analítica descritiva constitui o fundamento indispensável de qualquer avaliação benefício-risco^2^.

Entre as principais estruturas analíticas descritivas para avaliação benefício-risco, destacam-se: a “Benefit-Risk Action Team (BRAT)”; a “Problem, Objectives, Alternatives, Consequences, Trade-offs, Uncertainty, Risk, and Linked decisions (PrOACT-URL)” e a “US Food and Drug Administration - Benefit-Risk Framework (FDA BRF)”^3^^), (^^4^. A PrOACT-URL e a FDA BRF foram desenvolvidas por agências regulatórias. A European Medicines Agency (EMA), por exemplo, é responsável pela autorização final de comercialização de produtos medicinais para a União Europeia. Somente após essa autorização é que se inicia o processo de avaliação de tecnologias em saúde.

### Benefit-Risk Action Team (BRAT)

A estrutura analítica BRAT foi desenvolvida pela Pharmaceutical Research and Manufacturers of America (PhRMA) em colaboração com diversas empresas associadas^5^. Ela foi criada com base em revisão de literatura sobre os métodos existentes para avaliação benefício-risco, a qual evidenciou que nenhum dos métodos disponíveis incluía todos os critérios desejáveis - facilidade de uso, capacidade de atualização, integração de múltiplos benefícios e riscos, flexibilidade para aplicação em diversas áreas terapêuticas e a habilidade de incorporar as preferências das partes interessadas, como pacientes, médicos e reguladores^6^.

A BRAT foi desenvolvida com o propósito de aprimorar a avaliação benefício-risco e recomendar uma abordagem transparente e sistemática para apoiar a tomada de decisão. A estrutura analítica avalia separadamente os benefícios e os riscos, o que facilita sua comparação e evita a síntese dos dados em modelos estatísticos complexos, que podem ser de difícil compreensão para os leitores.

Entre 2008 e 2009, a BRAT foi aprimorada em colaboração com epidemiologistas, testada em casos simulados e avaliada por especialistas regulatórios e clínicos. Desenvolvida especificamente para avaliação benefício-risco de produtos farmacêuticos, a estrutura analítica fundamenta-se em metodologias consolidadas, como revisões sistemáticas de evidências e de metanálises e análise de decisão multicritérios^7^.

A BRAT apresenta as seguintes características: enfatiza a construção da árvore de valor, seleção e preparação de dados; avalia benefícios e riscos separadamente, não integrados na estrutura; pode incorporar a ponderação de resultados, sem se concentrar no cálculo de pontuações de benefício-risco geral^6^^), (^^7^^), (^^8^.

Ela é aplicada na tomada de decisão e na comunicação da avaliação benefício-risco entre as empresas farmacêuticas e os órgãos regulatórios por meio de um processo realizado em seis etapas^6^^), (^^7^^), (^^8^ (Tabela 1).

**Tabela 1:** Etapas da estrutura analítica benefit-risk action team (BRAT)

Etapa	Descrição
**1. Defina o contexto da decisão**	• Defina o medicamento, dose, formulação, indicação, população de pacientes, comparador(es), horizonte temporal para os desfechos e a perspectiva dos tomadores de decisão (regulador, pagador, paciente ou médico).
**2. Identifique os desfechos**	• Selecione todos os desfechos importantes e crie a árvore de valor inicial.
	• Defina um conjunto preliminar de medidas de desfecho para cada um.
	• Documente a justificativa para os desfechos incluídos/excluídos.
**3. Identifique as fontes de dados e extraia os dados**	• Determine e documente todas as fontes de dados (por exemplo, ensaios clínicos, estudos observacionais). Extraia todos os dados relevantes para a tabela de fontes de dados, incluindo referências detalhadas e quaisquer anotações, para ajudar nas interpretações subsequentes e criar medidas sumárias.
**4. Personalize a estrutura analítica**	• Modifique a árvore de valor com base na revisão adicional dos dados e na expertise clínica.
	• Refine as medidas de desfecho. Isso pode incluir ajustes nos desfechos que não são considerados relevantes para uma avaliação específica de benefício-risco ou que variam em relevância conforme o grupo de partes interessadas.
**5. Avalie a importância dos desfechos**	• Aplique ou avalie qualquer ranqueamento ou ponderação da importância dos desfechos para os tomadores de decisão ou outras partes interessadas.
**6. Apresente e interprete as principais métricas de benefício-risco**	• Resuma os dados em apresentações tabulares e gráficas para ajudar na revisão e interpretação.
	• Questione as medidas sumárias, revise as fontes de dados e identifique e preencha quaisquer lacunas de informação.
	• Interprete as informações resumidas.

### Problem, Objectives, Alternatives, Consequences, Trade-offs, Uncertainty, Risk and Linked decisions (PrOACT-URL)

A PrOACT-URL foi desenvolvida pela EMA em colaboração com a London School of Economics e a University of Groningen, no âmbito do EMA Benefit-Risk Methodology Project, entre 2010 e 2011^9^. Esse projeto revisou os métodos reconhecidos para avaliação benefício-risco e aqueles apresentados nos relatórios do Committee for Medicinal Products for Human Use (CHMP), com a sugestão da incorporação de critérios e de diretrizes específicas sobre benefícios e riscos, bem como o desenvolvimento de metodologias para análise sistemática do balanço benefício-risco^9^.

A PrOACT-URL segue a metodologia de tomada de decisão PrOACT, desenvolvida por cientistas da teoria da decisão^10^. Foi desenvolvida para melhorar a transparência, a consistência e a comunicação da avaliação benefício-risco, sendo especificamente direcionada para a tomada de decisões regulatórias sobre produtos medicinais^9^.

Sua abordagem qualitativa é suficiente para a maioria dos casos. Em situações mais complexas, no entanto, sua estrutura tem sido usada como base para outras metodologias^11^, porque permite a integração com outras metodologias quantitativas, como modelos de análise de decisão e análise de decisão multicritérios^12^.

A PrOACT-URL permite o uso de diferentes julgamentos e de fontes por diferentes partes interessadas, como desenvolvedores de medicamentos, órgãos regulatórios, órgãos de avaliação de tecnologias em saúde, prescritores e pacientes^13^. A flexibilidade dela é tal que sua implementação na avaliação benefício-risco pode se estender por todas as etapas do ciclo de vida de uma tecnologia em saúde, o que evidencia sua aplicabilidade ao campo da avaliação de tecnologias em saúde^3^. Um modelo quantitativo desenvolvido por um regulador pode ser reutilizado pelo órgão de avaliação de tecnologias em saúde, que pode adicionar critérios de custo e QALY, além de calcular a razão de custo-efetividade incremental^14^.

A PrOACT-URL^13^^), (^^15^ é realizada em 12 etapas (Tabela 2). A etapa seis sugere a criação de uma tabela de efeitos, que apresenta todos os efeitos favoráveis e desfavoráveis que os avaliadores consideram influenciar o balanço entre benefícios e riscos, juntamente os comentários sobre as incertezas que, na visão dos avaliadores, persistem na avaliação^13^.

**Tabela 2:** Etapas da estrutura analítica problem, objectives, alternatives, consequences, trade-offs, uncertainty, risk and linked decisions (PrOACT-URL)

Etapa	Descrição
**Problema**	
1. Determine a natureza do problema e seu contexto	• A tecnologia (por exemplo, novo medicamento ou biológico, dispositivo, genérico, comercializado).
	• Indicação(ões) de uso.
	• A área terapêutica e a epidemiologia da doença.
	• A necessidade médica não atendida, gravidade e morbidade da condição, população afetada, preocupações de pacientes e médicos, período para os desfechos de saúde.
	• O problema de decisão (o que deve ser decidido e por quem, por exemplo, indústria, regulador, prescritor, paciente).
2. Estruture o problema	• Se este é principalmente um problema de incerteza, ou de múltiplos objetivos conflitantes, ou uma combinação dos dois (por exemplo, progressão temporal dos estados de saúde).
	• Os fatores a serem considerados na resolução do problema (por exemplo, desenho do estudo, fontes e adequação dos dados, epidemiologia da doença, presença de tratamentos alternativos).
**Objetivos**	
3. Estabeleça objetivos que indiquem os propósitos gerais a serem alcançados	• O objetivo (por exemplo, avaliar o balanço benefício-risco, determinar quais informações adicionais são necessárias, avaliar a mudança no balanço benefício-risco, recomendar restrições).
4. Identifique critérios para: a) Efeitos favoráveis; b) Efeitos desfavoráveis	• Um conjunto completo de critérios que inclua os efeitos favoráveis e desfavoráveis (por exemplo, desfechos, estados de saúde relevantes, resultados clínicos). Uma definição operacional para cada critério juntamente com uma escala de medição com dois pontos definidos para abranger a gama de desempenho das alternativas (não apenas medidas de tendência central relatadas, mas também intervalos de confiança). Considerações sobre a relevância clínica dos critérios - alguns são de maior preocupação para os tomadores de decisão do que outros.
**Alternativas**	
5. Identifique as opções a serem avaliadas em relação aos critérios	• Pré-aprovação: dosagem, momento do tratamento, droga vs. placebo e/ou comparador ativo; a decisão ou recomendação necessária (por exemplo, aprovar/desaprovar, restringir, retirar).
	• Pós-aprovação: não fazer nada, limitar duração, restringir indicação, suspender.
**Consequências**	
6. Descreva como as alternativas se comportam para cada um dos critérios, ou seja, as magnitudes de todos os efeitos, gravidade e a incidência de todos os efeitos.	• As consequências separadamente para cada alternativa em cada critério (por exemplo, efeitos de eficácia e segurança que são clinicamente relevantes, desfechos de saúde positivos e negativos), resumidas em uma tabela de efeitos com alternativas nas colunas e critérios nas linhas. Descrições qualitativas e quantitativas dos efeitos em cada célula, incluindo resumos estatísticos com intervalos de confiança e referências aos dados de origem, gráficos e diagramas.
**Trade-offs**	
7. Avalie o balanço entre os efeitos favoráveis e desfavoráveis	• O julgamento sobre o balanço benefício-risco e a justificativa para o julgamento.
**Até essa etapa, apenas questões relacionadas aos efeitos favoráveis e desfavoráveis, e seu balanço, foram consideradas. As próximas três etapas são relevantes para considerar como o balanço benefício-risco é afetado ao levar em conta as incertezas.**	
**Incerteza**	
8. Relate a incerteza associada aos efeitos favoráveis e desfavoráveis.	• A base e a extensão da incerteza além das probabilidades estatísticas (por exemplo, possíveis vieses nos dados, solidez e representatividade dos ensaios clínicos, potencial para efeitos adversos não observados).
9. Considere como o balanço entre efeitos favoráveis e desfavoráveis é afetado pela incerteza.	• A extensão em que o balanço benefício-risco na etapa sete é reduzido ao considerar todas as fontes de incerteza, para fornecer um balanço benefício-risco, e as razões para a redução.
**Tolerância ao risco**	
10. Avalie a importância relativa da atitude do tomador de decisão em relação ao risco para este produto.	• Qualquer consideração que possa ou deva afetar a atitude do tomador de decisão em relação ao risco para este produto (por exemplo, status de medicamento órfão, população especial, necessidade médica não atendida, plano de gerenciamento de risco).
11. Relate como isso afetou o balanço relatado na etapa nove.	• A base para a decisão do tomador de decisão sobre o quão tolerável o balanço benefício-risco é considerado (levando em conta as opiniões das partes interessadas sobre o risco).
**Decisões interligadas**	
12. Considere a consistência desta decisão com decisões passadas semelhantes e avalie se tomar esta decisão pode impactar decisões futuras.	• Como esta decisão, e os julgamentos de valor e dados nos quais ela se baseia, podem estabelecer um precedente ou tornar decisões semelhantes no futuro mais fáceis ou mais difíceis.

É importante ressaltar que ambas as estruturas analíticas, BRAT e PrOACT-URL, têm como base princípios bem estabelecidos de análise de decisão^6^. As raízes comuns dos métodos resultam em muitas semelhanças e uma relação estreita entre eles. A principal diferença é que os dois últimos passos da estrutura analítica ProACT-URL consideram questões mais amplas, como atitudes de risco do tomador de decisão e consistência com decisões anteriores semelhantes, questões que não são partes formais da BRAT^15^.

### US Food and Drug Administration - Benefit-Risk Framework (FDA BRF)

A FDA BRF foi desenvolvida pela FDA (Food and Drug Administration) e sua implementação teve início em 2013^12^. Essa estrutura analítica fornece uma abordagem estruturada para avaliação benefício-risco de medicamentos e de produtos biológicos, com a contemplação de questões relevantes para a tomada de decisão regulatória e para a comunicação interna à FDA e ao público^16^.

Na FDA, a avaliação benefício-risco não termina com a aprovação de um medicamento, mas continua ao longo de todo o seu ciclo de vida do medicamento, pois os benefícios e os riscos podem mudar com o tempo, a partir de novas informações sobre sua eficácia ou segurança. No período pós-comercialização, o balanço benefício-risco é reavaliado com base na literatura científica, estudos pós-marketing, relatos de eventos adversos, erros de medicação, qualidade do produto e dados de experiência do paciente^16^.

A FDA reconhece a importância de incorporar a contribuição dos pacientes no desenvolvimento dos medicamentos e nas decisões regulatórias, com a consideração das suas experiências e das suas perspectivas essenciais para compreender o impacto da doença, os benefícios mais relevantes e a aceitação dos riscos ao longo do ciclo de vida do medicamento^16^.

A FDA BRF^16^ oferece diretrizes claras e de fácil aplicação. Analisa as dimensões de decisão, que inclui a análise da condição, as opções de tratamento atuais, os benefícios, os riscos e o gerenciamento desses riscos. Considera-se também as evidências e as incertezas relacionadas a esses fatores, que levam às conclusões e às razões subjacentes, ou seja, às implicações para a tomada de decisão. (Tabela 3)

**Tabela 3:** Descrição dos fatores de decisão, evidências, incertezas, conclusões e razões para avaliação benefício-risco, por meio da estrutura analítica US food and drug administration - benefit-risk framework (FDA BRF)

Dimensões	Considerações importantes	Conclusões e razões
**Análise da condição**	• Contexto de uso para a indicação proposta: uso médico pretendido, população-alvo de pacientes, aspectos da condição que o tratamento busca abordar;	• Conclusões (implicações para decisão)
	• Aspectos da condição indicada mais relevantes;	
	• Implicações de saúde pública da doença;	
**Opções de tratamento atuais**	• Compreensão do tratamento atual aprovado pelo FDA e do padrão de cuidado;	• Conclusões (implicações para decisão)
	• Eficácia e segurança de outras intervenções disponíveis para a população alvo;	
	• Necessidade médica dos pacientes por uma nova terapia	
**Benefício**	• Pontos fortes/limitações dos ensaios clínicos, incluindo o delineamento, e suas possíveis implicações para avaliação da eficácia do medicamento;	• Conclusões (implicações para decisão)
	• Relevância clínica dos desfechos do estudo: capacidade de medir ou prever resultados clínicos importantes para os pacientes;	
	• Descrição dos benefícios clínicos, incluindo, mas não se limitando a:	
	• Natureza do efeito (por exemplo, sobrevida, desaceleração da progressão da doença, redução de desfechos graves, redução de sintomas, relevância do benefício sintomático para os pacientes)	
	• Magnitude do efeito e incerteza associada (por exemplo, intervalo de confiança), acompanhadas de uma interpretação de sua relevância clínica	
	• Distribuição dos efeitos do tratamento na população do ensaio clínico (por exemplo, presença de pacientes que experimentam um benefício mais substancial, como sobrevida prolongada ou melhora acentuada dos sintomas, mesmo quando a resposta média é modesta)	
	• Evolução temporal e durabilidade do efeito	
	• Benefício atribuído ao medicamento quando estudado em combinação com outras terapias	
	• Subpopulações definidas que alcançam uma maior magnitude de benefício	
	• Benefício para uma subpopulação com necessidade médica não atendida específica (por exemplo, pacientes que não responderam adequadamente às terapias disponíveis)	
	• Generalização dos benefícios demonstrados para todas as populações que provavelmente receberão o medicamento (por exemplo, pacientes idosos ou com comorbidades não amplamente estudadas nos ensaios clínicos)	
	• Características importantes do medicamento (por exemplo, regime de dosagem menos oneroso, forma de preparo da dose ou via de administração).	
**Risco e gerenciamento de risco**	• Pontos fortes e limitações das evidências relacionadas à segurança, e suas possíveis implicações para a avaliação dos riscos do medicamento (por exemplo, devido ao tamanho limitado do banco de dados e/ou à duração da exposição, ou à ausência de subpopulações importantes).	• Conclusões (implicações para decisão)
	• Eventos adversos observados ou sinais de segurança e sua relevância clínica, incluindo:	
	• Gravidade do evento adverso, probabilidade de ocorrência, reversibilidade e estimativa da magnitude do efeito e sua incerteza (por exemplo, intervalo de confiança);	
	• Capacidade de prever, monitorar e/ou prevenir o evento adverso;	
	• Impacto dos eventos adversos na tolerabilidade e/ou adesão ao medicamento, e as possíveis consequências.	
	• Nível de certeza quanto à associação causal entre a exposição ao medicamento e o risco.	
	• Impacto potencial de questões relacionadas à qualidade do produto que possam afetar negativamente a segurança ou eficácia do medicamento, incluindo considerações de qualidade que precisariam ser monitoradas após a comercialização.	
	• Diferenças esperadas de segurança no período pós-comercialização, em comparação com o contexto dos ensaios clínicos (por exemplo, devido à menor probabilidade de monitoramento adequado ou ao uso em pacientes com maior risco de eventos adversos).	
	• Potencial de uso indevido ou exposição acidental, e as consequências adversas associadas.	
	• Eficácia provável das abordagens propostas para o gerenciamento desses riscos.	
**Conclusões sobre o balanço benefício-risco**		
• Conclusões gerais sobre a qualidade e a robustez das evidências, bem como sobre as incertezas remanescentes em relação aos benefícios e riscos.		
• Influência do contexto terapêutico na avaliação dos benefícios, riscos e incertezas.		
• Importância relativa dos benefícios e riscos na população-alvo geral, considerando também as perspectivas dos pacientes.		
• Evolução temporal dos benefícios e riscos (por exemplo, levando em conta eventos adversos que podem ocorrer logo após o início do tratamento em contraste com benefícios que podem levar anos para se manifestar).		
• Capacidade de pacientes e profissionais de saúde de avaliar claramente os benefícios ou riscos do medicamento (por exemplo, por meio da avaliação do alívio dos sintomas ou do monitoramento de alterações em biomarcadores), contribuindo assim para decisões terapêuticas informadas (por exemplo, descontinuar o medicamento se não houver resposta adequada).		

## Aplicação de estruturas analíticas descritivas para avaliação benefício-risco no contexto da avaliação de tecnologias em saúde

A utilização de estruturas analíticas descritivas em avaliação benefício-risco tem sido observada ao longo das três últimas décadas (2000, 2010, 2020), com vistas a aplicação de abordagens mais estruturadas, objetivas e transparentes, com consequente melhora na comunicação e na tomada de decisões^3^^), (^^11^^), (^^12^^), (^^17^. No contexto da avaliação de tecnologias em saúde, de acordo com uma revisão de escopo publicada em 2024, as estruturas analíticas descritivas mais citadas foram a PrOACT-URL e a BRAT. Já as mais recomendadas foram a PrOACT-URL e a FDA BRF^4^.

A BRAT, a PrOACT-URL e a FDA BRF compartilham características comuns, como praticidade, consistência, transparência, abordagem sistemática e contexto de decisão. Também compartilham a qualidade e a interpretação das evidências, a transparência da priorização e da ponderação de benefícios e de riscos e o monitoramento das incertezas. Finalmente, as três compartilham a avaliação, o resumo e a comunicação de dados e de informações relevantes por meio de ferramentas visuais como árvores de valor ou tabelas de efeitos, que serão discutidas no último artigo desta série^3^.

A FDA mantém o uso de uma estrutura analítica descritiva para avaliação benefício-risco, apesar de reconhecer o potencial dos métodos quantitativos. A EMA tem avançado na adoção de estruturas analíticas quantitativas^18^, que serão abordadas no próximo artigo desta série.

É preciso salientar que, nos casos em que uma nova tecnologia aumenta os benefícios e diminui os riscos, ou quando os riscos superam claramente os benefícios, a aplicação de uma estrutura analítica descritiva é suficiente para determinar o balanço benefício-risco dessa tecnologia^19^. Recentemente, o Grading of Recommendations Assessment, Development and Evaluation (GRADE) estabeleceu o uso de limiares de decisão que podem apoiar os julgamentos sobre o balanço entre os benefícios e os riscos (danos) na avaliação de tecnologias em saúde^20^, que serão apresentados no penúltimo artigo desta série.

No que se refere ao contexto brasileiro de avaliação de tecnologias em saúde, a implementação dessas estruturas analíticas pode representar uma oportunidade para qualificar ainda mais a deliberação em instâncias, como a Conitec, especialmente ao tornar mais explícitos os critérios e as incertezas envolvidos nas recomendações. Os desafios para tanto, contudo, incluem a necessidade de capacitação técnica, a adaptação às especificidades institucionais e regulatórias do Sistema Único de Saúde e a integração com os atuais fluxos de avaliação e de participação social.

As terapias gênicas e, em geral, as tecnologias destinadas ao tratamento das doenças raras são bons exemplos que podem ser objetos das capacitações técnicas. Essas tecnologias exigem uma atenção diferenciada das agências regulatórias e de avaliação de tecnologias em saúde, já que a avaliação benefício-risco tende a ser mais complexa e com elevado grau de incerteza decorrente da escassez de dados, do número reduzido de pacientes e de limitações metodológicas dos estudos disponíveis.

Recomenda-se que os estudos completos de avaliação de tecnologias em saúde considerem a inclusão de pelo menos uma estrutura analítica descritiva estruturada^17^, com vistas a apresentar de forma transparente os julgamentos de valor e a fundamentação que apoiaram a tomada de decisão, com garantia de que os principais aspectos do processo de avaliação não foram negligenciados. O balanço entre benefícios e riscos (danos) baseado em evidências também deve ser comunicado explicitamente nos processos decisórios.

## Data Availability

Os dados estão disponíveis no corpo do manuscrito.
